# Periprosthetic Fractures in Long Versus Short Proximal Femoral Nailing for Intertrochanteric Fractures: A 10-Year Single-Centre Cohort Study

**DOI:** 10.7759/cureus.32892

**Published:** 2022-12-23

**Authors:** Omer Nasim, Suraj Kohli, Boulos Eskander, Sameh Girgis, Michael Kent

**Affiliations:** 1 Trauma and Orthopaedics, University Hospitals Dorset, Poole, GBR; 2 Trauma and Orthopaedics, Salisbury NHS Foundation Trust, Salisbury, GBR; 3 Trauma & Orthopaedics, University Hospitals Dorset, Poole, GBR

**Keywords:** neck of femur fracture (nof), nof, elderly population, neck of femur fractures, fragility fracture, femur intertrochanteric fracture, periprosthetic fracture, proximal femoral nail, orthopaedics surgery

## Abstract

Background

Neck of femur (NOF) fractures, including intertrochanteric fractures, are common fragility fractures seen in the elderly population and are often amenable to fixation with a proximal femoral nail (PFN). However, there is conflicting evidence regarding the incidence of peri-prosthetic fractures with each device. Several studies from the 1990s and a recent meta-analysis have shown a higher incidence of peri-prosthetic fractures in the short PFN cohort. Other studies have shown a lower reoperation rate with short PFNs, and no statistically significant differences have been quoted in the rates of peri-prosthetic fractures in systematic reviews.

Aim

The purpose of this retrospective study, therefore, was to assess the peri-prosthetic fracture rate and failure rates of elderly neck of femur (NOF) fractures implanted with either a short or long proximal femoral nail (PFN).

Materials and methods

A retrospective study was conducted in a single orthopaedic department (University Hospital Dorset, Poole, GBR) using collected data on all extracapsular neck of femur fracture patients from the national hip fracture database (NHFD) from January 1, 2011, to December 23, 2021. The data collected included patient age, sex, the American Society of Anesthesiologists (ASA) type of neck of femur fracture, type of surgery performed, any further peri-prosthetic fractures, and time to re-operation in that subset of the group.

The implants used were the Stryker Gamma 3 Nail and the Smith Nephew (Trigen and Intertan). All patients were allowed to fully weight bear as tolerated and received both orthopaedic and elderly medical care. Failure was defined as a cut-out or implant fracture.

Results

From January 1, 2011, to December 23, 2021, there were 1010 extracapsular neck of femur fractures recorded on the National Hip Fracture Database (NHFD) treated with a PFN from the study centre. Of those patients, 11 had pathological fractures and were excluded. 649 patients had long PFNs, and 350 had short PFNs. Of the total of 999 patients, 254 (25%) were male and 745 (75%) were female. More than 80% of the patients in the sample were over the age of 75. The majority of patients in both groups had A1/A2 fractures (short 84.3%, long 49.1%).

The rate of periprosthetic fractures in the short PFNs was 1.71%, and the failure rate was 0.57%. The rate of periprosthetic fractures in the long PFNs was 0.62%, with a failure rate of 0.92%. The multi-nominal logistic regression model did not show statistically significant odds ratios (OR) for the following variables: long/short nails, male/female gender, age, ASA, or type of fracture. The female gender was associated with a higher risk of both periprosthetic fractures and failures (OR of 2.232 and 2.95), but this was not found to be statistically significant. Similarly, unstable A3 fractures had a much higher risk of failure (OR of 2.691) compared to periprosthetic fractures (OR of 0.985). However, this was not statistically significant.

Conclusion

Overall, this study has identified that in a patient population that is predominantly female and over the age of 75, the risk of periprosthetic fracture rate and the failure rate is similar in both the use of a short or a long PFN for intertrochanteric fractures.

## Introduction

Neck of femur (NOF) fractures, including intertrochanteric fractures, are common fragility fractures seen in the elderly population. Approximately 63000 NOFs occurred in the UK during 2020, with annual worldwide incidences set to rise to 6.36 million by 2050 [1,2]. With a 30-day mortality rate of 10% and a one-year mortality rate of 33%, optimisation of surgical and post-operative care is essential [3].

Intertrochanteric NOF fractures make up approximately one-third of these injuries and can be classified as stable (AO/OTA 31-A1 and A2) or unstable (31-A3) [4]. Surgical management of these injuries has sparked considerable debate and is often done with a sliding or dynamic hip screw or a proximal femoral nail (PFN). In recent years, there has been a change in the trend of surgical fixation, with PFNs becoming increasingly popular [5].

Intramedullary nails have been shown to be effective in treating unstable intertrochanteric and subtrochanteric fractures. They act as an intramedullary buttress and prevent medialisation of the femoral shaft [6]. Although both short and long proximal femoral nails (PFNs) are available for the treatment of intertrochanteric NOFs, short PFNs have been shown to have a shorter operating time, reduced blood loss, and fewer blood transfusions [7]. However, there is conflicting evidence regarding the incidence of periprosthetic fractures with each device. Several studies from the 1990s [8,9] and a recent meta-analysis have shown a higher incidence of periprosthetic fractures in the short PFN cohort [10]. Other studies have shown a lower reoperation rate with short PFNs [11,12], and no statistically significant differences have been quoted in the rates of periprosthetic fractures in systematic reviews [12].

The purpose of this retrospective study, therefore, was to assess the periprosthetic fracture rate and failure rates of elderly NOFs implanted with either a short or long PFN.

## Materials and methods

A retrospective study was conducted in a single orthopaedic department (University Hospital Dorset, Poole) using collected data on all extracapsular neck of femur fracture patients from the National Hip Fracture Database (NHFD) from January 1, 2011, to December 23, 2021. The data collected included patient age, sex, the American Society of Anaesthesiologists (ASA), type of neck or femur fracture, type of surgery performed, any further periprosthetic fractures, and time to reoperation in that subset of the group. The raw dataset was analysed to identify the cohort of patients with extracapsular neck of femur fractures who preoperatively met the National Institute for Health and Care Excellence (NICE) criteria for consideration of surgical fixation and if they were medically fit for anaesthesia and the procedure. Patients with pathological fractures were excluded. The follow-up period for patients was 120 days.

Antibiotics were given at induction for all cases, and all nails were inserted under fluoroscopic guidance. The implants used were the Stryker Gamma3 nail and the Smith-Nephew (Trigen and Intertan). All patients were allowed to fully weight bear as tolerated and received both orthopaedic and elderly medical care.

The electronic patient records (EPR) system (Crouch, 2020), which includes all outpatient clinic letters and inpatient discharge summaries as well as scanned inpatient medical and operative notes, was then reviewed for each patient to identify any follow-up and any other fractures reported in the following years. A comparison was made between the two cohorts with long nails and short nails to assess for significant differences regarding complication and revision surgery rates. The data was then further analysed to identify any specific patient factors related to an adverse outcome for either the proximal femoral short or long nail. Failure was defined as a cut-out or implant fracture.

Statistical analysis

Dichotomous variables were assessed using a chi-square test and a Student's t-test. A p-value of <0.05 was defined as significant. Univariate logistic regression was performed for each variable (age at surgery, ASA grade, sex, type of fracture, long or short nail). This was then followed by a multiple nominal logistical regression to assess each variable and their relationship to failure and periprosthetic rate. P-values were set to <0.05 for significance. Reference categories were stable fractures (A1/A2), long nails, and male gender.

Ethical statement

The data collected formed part of the study centre’s participation in the National Hip Fracture Database. There was no additional patient contact, and as such, this project was performed as a service evaluation without the need for formal ethical approval. The project was registered with the trust’s clinical audit department (Reg # 5372) and was conducted in accordance with the Declaration of Helsinki and the guidelines for good clinical practice.

## Results

Patient demographics

From January 1, 2011, to December 23, 2021, there were 1010 extracapsular neck of femur fractures recorded on the National Hip Fracture Database (NHFD) treated with a PFN from the study centre. Of those patients, 11 had pathological fractures and were excluded from the final analysis. 649 patients had long PFNs, and 350 had short PFNs. Of the total of 999 patients, 254 (25%) were male and 745 (75%) were female.

There was a similar distribution of patients in the male and female groups for ASAs 1-3; however, there was double the percentage of ASA 4s in the male group (17.3% vs. 8.9%). This is outlined in Table 1. The majority of patients in both groups were ASA 3 (65%).

**Table 1:** ASA grade classification and breakdown by gender ASA: The American Society of Anaesthesiologists

ASA grade classification			Total
Variable	Female	Male	
1	6(0.8%)	3(1.2%)	9(0.9%)
2	186(25.0%)	41(16.1%)	227(23.6%)
3	487(65.4%)	166(65.4%)	653(65.4%)
4	66(8.9%)	44(17.3%)	110(11%)
Total	745(74.5%)	254(25.4%)	999

There was no statistically significant increase in the number of males in both the short and long PFN groups (Table 2). The majority of patients who had grade A1/A2 fractures had fixations with short PFNs (84.3%) and with long PFNs (49.1%). Of the ones with grade A3 fractures, 15.1% had fixations with short PFNs, while 30.8% had fixations with long PFNs. Of those with subtrochanteric fractures, 0.6% had fixations with short PFNs, and 20% had fixations with long PFNs.

**Table 2:** Patient demographics comparison for long and short PFN cohorts PFN: proximal femoral nail

Variable	Long PFN (N=649)	Short PFN (N=350)	p-value
Sex (Male)	172	82	0.287

More than 80% of the patients in the sample were over the age of 75. Only four patients (0.5%) were less than 50 (Table 3).

**Table 3:** Age distribution in the sample population

Age at surgery	
Age range (in years)	Frequency
<50	4 (0.5%)
50-65	39 (3.9%)
65-75	130 (13.0%)
75-85	327 (32.7%)
85<	499 (49.9%)
Total	999

The majority of patients in both groups had A1/A2 fractures (short 84.3%, long 49.1%). With regard to subtrochanteric fractures in male patients, none were treated with short nails; only two cases of female patients with subtrochanteric fractures were treated with short nails. There was a similar distribution of fracture types by gender in the short and long nail cohorts (Table 4).

**Table 4:** Breakdown of fracture type distributions by gender and mode of fixation IM: intramedullary

Fracture type	IM nail (long)		IM nail (short)	
	Female	Male	Female	Male
Intertrochanteric - grade A1/A2	221 (46.3%)	98 (57.0%)	219 (81.7%)	76 (93.7%)
Intertrochanteric - grade A3	157 (32.9%)	43(25%)	47 (17.5%)	6 (7.3%)
Subtrochanteric	99 (20.8)	31(18.0)	2 (0.75%)	0 (0%)
Grand total	477 (100%)	172 (100%)	268 (100%)	82 (100%)
	IM nail (long)		IM nail (short)	
	Female	Male	Female	Male
Intertrochanteric - grade A1/A2	221 (46.3%)	98 (57.0%)	219 (81.7%)	76 (93.7%)
Intertrochanteric - grade A3	157 (32.9%)	43(25%)	47 (17.5%)	6 (7.3%)
Subtrochanteric	99 (20.8)	31(18.0)	2 (0.75%)	0 (0%)
Grand total	477 (100%)	172 (100%)	268 (100%)	82 (100%)

The rate of periprosthetic fractures in the short PFNs was 1.71%, and the failure rate was 0.57% (Table 5). The rate of periprosthetic fractures in the long PFNs was 0.62% with a failure rate of 0.92%. There were no short PFN failures in the male cohort.

**Table 5:** Patient demographics comparison for long and short PFN cohorts for periprosthetic fractures PFN: proximal femoral nail

Row labels	Failure	Periprosthetic fracture	Grand total
Female	7	9	16
Long PFN	5	3	8
Short PFN	2	6	8
Male	1	1	2
Long PFN	1	1	2
Short PFN	0	0	0
Grand total	8	10	18

The results of the multinominal logistic regression model did not show statistically significant odds ratios for the following variables: long/short nails, male/female gender, age, ASA, or type of fracture. The female gender was associated with a higher risk of both periprosthetic fractures and failures (OR 2.232 and 2.95), but this was not found to be statistically significant. Similarly, unstable A3 fractures had a much higher risk of failure (OR 2.691) compared to periprosthetic fractures (OR 0.985), but this was not statistically significant.

**Table 6:** Odds ratios for multinomial logistic regression assessing failure and periprosthetic fracture rate ASA: The American Society of Anaesthesiologists

Failure or peri-prosthetic fracture?				95% Confidence interval for Exp (B)	
		p-values	OR	Lower bound	Upper bound
Failure					
	Age at surgery	0.432	0.749	0.365	1.539
	ASA grade	0.446	0.639	0.201	2.026
	Female	0.461	2.232	0.265	18.814
	Male	.	.	.	.
	Subtrochanteric	0.868	1.224	0.113	13.29
	A3	0.22	2.691	0.553	13.085
	A1/A2	.	.	.	.
	Short nail	0.775	0.779	0.14	4.33
	Long nail	.	.	.	.
Periprosthetic fracture					
	Age at surgery	0.906	1.046	0.494	2.216
	ASA grade	0.442	0.659	0.228	1.907
	Female	0.31	2.95	0.366	23.783
	Male	.	.	.	.
	Subtrochanteric	0.687	0.636	0.07	5.772
	A3	0.984	0.985	0.231	4.201
	A1/A2	.	.	.	.
	Short nail	0.642	0.712	0.17	2.983
	Long nail	.	.	.	.
a The reference category is None.					

## Discussion

PFNs are a common fixation method for intertrochanteric NOFs. Concerns were raised initially as first-generation short PFNs had a significantly increased risk of periprosthetic fractures [13]. Preference was therefore given to longer intramedullary devices. This study has shown no increased risk of periprosthetic fracture or failure rate in a predominantly over-75 patient cohort. Additionally, there was no association between type of fracture, ASA grade, gender, age, or type of fixation method and periprosthetic fracture or failure rates.

The female gender was associated with a higher risk of both periprosthetic fractures and failure rates, but this was not found to be statistically significant (OR 2.232 and 2.95, respectively). Our patient population had a significantly higher percentage of females (75%), which is similar to quoted epidemiological incidence rates and is likely representative of the patient cohort an acute hospital may treat [14]. Bone density on average is much lower in elderly females compared to males, with an annual loss of density being 0.5-0.7% greater in females at age 60 and over [15]. Therefore, with osteoporotic bone, the fixation of the fracture may not be as sound, leading to a potentially higher risk of periprosthetic fractures or failure.

This study showed a periprosthetic fracture rate of 1.71% for short PFNs and 0.62% for long PFNs. This is similar to other studies, where periprosthetic rates ranged from 0-2.7% for short nails and 0-1.5% for long nails [16, 17, 18]. Given the complexity of long-nail periprosthetic fracture management, short nails could be viewed as more favourable. For a short nail, periprosthetic fractures can be treated with the application of a longer intramedullary device. However, for long nails, periprosthetic fracture management is more complex, with plate/nail hybrid constructs, long nail removal, or distal femoral replacements. Given the elderly and comorbid population of these fractures, complex revision surgery will require a longer anaesthetic time, more blood loss, and likely a higher mortality rate.

This study also showed a failure rate of 0.97% for short PFNs and 0.52% for long PFNs. This is lower than other studies' estimates of 4%-4.1% for short nails and 3.2%-6.4% [11,19]. This may be due to the length of follow-up of our sample at 120 days compared to two years, which may not have captured all of the failures [11,19].

Short or long?

There are clinical, biomechanical, and economical factors that should be taken into consideration when choosing an implant for the fixation of NOF fractures. Short PFNs have been shown to have a shorter operating time, reduced blood loss, and fewer blood transfusions, and are therefore advantageous as patients with these injuries are often elderly and comorbid [7].

Long PFNs span the length of the femur and should theoretically have a greater mechanical advantage over short nails. However, biomechanical studies comparing short and long PFNs showed no statistically significant differences in the mechanical failure of these implants [20,21]. Long nails also have the complication of anterior cortical impingement and a 1.5% incidence rate of iatrogenic insertion fractures [16]. Additionally, from an economical point of view, they can be 45% more expensive than short nails [16].

Surgeon or centre preference and familiarity with implants are important factors in the decision-making process for the management of these injuries. However, there is clinical, economical, and biomechanical evidence that short PFNs may be preferable to long PFNs in treating intertrochanteric NOF fractures.

## Conclusions

Overall, this study has identified that in a patient population that is predominantly female and over the age of 75, the risk of periprosthetic fracture rate and the failure rate is similar in both the use of a short or a long PFN for intertrochanteric fractures.
